# Emerging precision diagnostics in advanced cutaneous squamous cell carcinoma

**DOI:** 10.1038/s41698-022-00261-z

**Published:** 2022-03-23

**Authors:** Glenn Geidel, Isabel Heidrich, Julian Kött, Stefan W. Schneider, Klaus Pantel, Christoffer Gebhardt

**Affiliations:** 1 Department of Dermatology and Venereology, University Medical Center Hamburg-Eppendorf, Hamburg, Germany; 2 University Skin Cancer Center Hamburg, University Comprehensive Cancer Center Hamburg, University Medical Center Hamburg-Eppendorf, Hamburg, Germany; 3 Institute of Tumor Biology, University Medical Center Hamburg-Eppendorf, Hamburg, Germany

**Keywords:** Squamous cell carcinoma, Diagnostic markers, Prognostic markers

## Abstract

Advanced cutaneous squamous cell carcinoma (cSCC) encompasses unresectable and metastatic disease. Although immune checkpoint inhibition has been approved for this entity recently, a considerable proportion of cases is associated with significant morbidity and mortality. Clinical, histopathological, and radiological criteria are used for current diagnostics, classification, and therapeutic decision-making. The identification of complex molecular biomarkers to accurately stratify patients is a not yet accomplished requirement to further shift current diagnostics and care to a personalized precision medicine. This article highlights new insights into the mutational profile of cSCC, summarizes current diagnostic and therapeutic standards, and discusses emerging diagnostic approaches with emphasis on liquid biopsy and tumor tissue-based analyses.

## Introduction

Cutaneous squamous cell carcinoma (cSCC) is the second most frequent skin tumor arising from malignant progression of keratinocytes^1^. Cumulative UV-exposure of the skin remains a key factor for the development of invasive cSCC, whilst changing demographics towards aging populations contributes to a consistently rising - though hardly subsumable - incidence of disease worldwide. The relevance of invasive cSCC is denoted by a range of twice the incidence of melanoma in European Caucasians of up to 10 times in fair-skinned populations in Australia^2^. Recent findings also suggest a sex-biased susceptibility of cSCC highlighting a certain protective role of female immunity^3^.

Ultraviolet radiation (UVR) is a central factor of carcinogenesis. It results in a high tumor mutational burden, which may challenge advances in precision medicine in advanced cSCC. In the last years, many studies aimed at the characterization of the mutational landscape of cSCC. They provided insight into the mutanome of advanced cSCC populations, which may help to point out new therapeutic targets and resistance mechanisms. Both tissue- and liquid biopsy-based approaches are under investigation for biomarker identification. A thorough understanding of cSCC mutagenesis and its mutational landscape will be helpful in the development and application of these techniques in advanced cSCC.

In this perspective, we therefore first summarize current knowledge on cSCC mutagenesis and mutational landscape presumably relevant for precision medicine approaches. We then discuss current diagnostics and treatment of advanced cSCC with perspectives of molecular biological tissue- and liquid biopsy-based techniques towards precision medicine.

## Biology of CSCC development and progression

### CSCC mutagenesis

Chronic cumulative exposure to UVR, predominantly UVA- and UVB-light, constitutes the most important risk factor for cSCC. The UV-mediated induction of photoproducts like cyclobutane pyrimidine dimers (CPDs) or pyrimidine-6,4-pyrimidone dimers (6-4PPs), among other alterations, may result in typical UV fingerprint mutations, if not repaired by nucleotide excision repair (NER)^4^. Besides a direct mutagenic effect, UVR may contribute to tumor development and progression via enhancement of multiple other processes such as local inflammation and immunosuppression^5^.

Understanding of the genomic correlates of a clinical multi-step evolvement of invasive cSCC from sun-exposed skin via “precancerous” lesions like actinic keratoses (AK) or Bowen’s disease (BD) and in situ cSCC (cSCCis) has been subject of many studies (Table 1). Despite all associations, it remains unclear which clone will eventually progress to cSCC or metastatic disease and what the specific underlying molecular mechanism is^6^. What we do know is that sun-exposed skin already is composed of thousands of mutants within every cell and a high clonal heterogeneity, in 18–32% exhibiting clones with about a handful driver mutations known for cSCC, but phenotypically not showing malignant transformation^7^.

**Table 1:** Reports on somatic mutational landscape in human cSCC (selection).

Reference	Samples included (total number)	Focus of study	Contribution to the field
Chang et al.^15^	cSCC (105)	Meta-analysis of 10 different studies	Up-to-date most comprehensive list of 30 bona fide driver genes with consideration of subgroups (IS, azathioprine, RDEB)
Thomson et al.^14^	AK IC (14), AK IS (23)	Specific genomic alterations	Azathioprine mutational signature (Inman et al.^13^) Dysregulation, increasing from AK to cSCC development (Cammareri et al.^83^) Similar TMB, patterns of driver genes and CNA between AK and cSCC 22 mutations occuring early in AK 22 mutations occuring late in AK CDKN2A is an early event in AK pathogenesis
Jones et al.^21^	Advanced cSCC (7)	Targetable mutations	ERBB3 mutation; addition of lapatinib results in stabilization of disease of respective patient
Lobl et al.^84^	High-risk cSCC (10), metastatic cSCC (10)	High-risk vs. metastatic cSCC	Wnt signaling pathway alteration confined to metastatic samples Mutations restricted to high-risk and metastatic cSCC CDH1 driver mutation in metastatic cohort
Lazo de la Vega et al.^85^	Cutaneous: AK (8), cSCCis (30), cSCC (18); Ocular: CIN (2), CIS (20), SCC (21)	Ocular vs. cutaneous SCC	Similar spectrum of genetic changes of precursor and invasive lesions from ocular vs. cutaneous cSCC
Zilberg et al.^86^	High-risk cSCC from head and neck region, treatment-naïve (10)	Suitability for targeted therapies	Predominance of loss-of-function TSG mutations Secondary or resistance mutations in 70% of cohort, which are known to develop in response to stressors (chemotherapy, targeted therapy), such as Ras, KIT, PDGFRA, or ABL1 mutations Some tumors exhibited targetable Ras (50%) and EGFR mutations (40%)
Inman et al.^13^	cSCC WD (20), cSCC MD/PD (20)	WD vs. MD/PD	CDKN2A gate keeper mutation and early event signature associated with azathioprine exposure; duration of exposure correlates with signature intensity NOTCH1/2, TP53, CDKN2A among most frequent alterations TGFβ alteration enriched in MD/PD subgroup 8 mutations occuring early in cSCC ATP1A1 associated with WD; GRHI2 associated with MD/PD
Zilberg et al.^87^	High-risk cSCC from head and neck region (24)	Clinical relevance	FGFR2 exclusively in PNI MLH1 exclusively in young patients <45 years
Yilmaz et al.^88^	cSCC (10), metastatic cSCC (18)	Metastatic vs. primary cSCC	Higher mutation frequencies of TP53 and KMT2D in metastatic cSCC No difference in KMT2C alterations No KNSTRN mutation Mutations in epigenetic and chromatin regulators may be associated with metastatic cSCC
Cammareri et al.^83^	Vemurafenib-associated lesions (39, *n* = 7), sporadic cSCC (31 WD, 31 MD, 29 PD), sporadic cSCC with matched perilesional skin (7)	Mutations facilitating carcinogenesis	TGFβ-receptor mutations occurred in 43% of sporadic and 28% of vemurafenib induced skin lesions. Loss of function is a common event in cSCC TGFβ-receptor mutations are early occuring events and candidate driver events
Chitsazzadeh et al.^89^	Normal skin/ AK/ cSCC (*n* = 12)	Targetable mutations	High degree of mosaicism across exome of sun-exposed perilesional skin Identification of candidate transcriptional drivers Key genomic changes supposedly appear in normal skin to AK transition
Martincorena et al.^7^	Sun-exposed (234, *n* = 4)	Sun-exposed skin	18–32% of sun-exposed skin harbors “driver mutations” known for cSCC Sun-exposed skin may harbor clones with 2–3 driver mutations not showing malignant transformation Identification of certain frequent mutations in lower levels in sun-exposed skin already No CDKN2A mutation detected Clonal heterogeneity, mutational burden 2–6 mutations/Mb/cell
Li et al.^90^	cSCC lymph node metastases (29)	Metastatic cSCC	Clinically targetable BRAF, FGFR3, PIK3CA, EGFR mutations Similarity of genomic alterations to previous reports 45% Ras/RTK/PI3K pathway mutations, correlating with worse PFS (not EGFR/ERBB4 mutation) Chromatin remodeling mutation correlate with worse PFS No KNSTRN mutation
Schwaederle et al.^91^	Different SCC entities (361; among these 36 cSCC); non-SCC (277)	SCC vs. non-SCC	8 gene “squamousness-signature” of SCC compared to non-SCC 2 SCC subgroups based on TP53 and PIK3CA mutation frequency
Pickering et al.^11^	Aggressive cSCC from head and neck region (39)	Driver mutations, novel targets	KMT2C mutations associated with poor outcome and increased bone invasion 8 significantly mutated genes common to HNSCC AJUBA mutation correlates with depth of invasion NOTCH2 mutation correlated with PNI Potentially targetable oncogenic events in STK19
South et al.^92^	Sporadic cSCC (132), vemurafenib-associated lesions (39), normal skin adjacent to cSCC (10)	Driver genes in sporadic/kinase-inhibitor induced cSCC	NOTCH1 mutations are among the most frequent and appear early, already in phenotypically normal skin
Lee et al.^93^	cSCC and matched adjacent skin (100); cSCC (38), AK (27)	KNSTRN mutations in AK and cSCC	KNSTRN mutations occurred in 19% of cSCC KNSTRN mutations are an early event (detection in AK at similar frequencies) Correlation of KNSTRN (8p.Ser24Phe) mutations with aneuploidy and supposedly more aggressive disease

*cSCC* cutaneous squamous cell carcinoma, *IS* immunosuppressed, *RDEB* recessive dystrophic epidermolysis bullosa, *IC* immunocompetent; *AK* actinic keratosis, *TMB* tumor mutational burden, *TGFβ* tumor growth factor beta, *cSCCis* in situ cSCC, *WD* well-differentiated, *MD* medium-differentiated, *PD* poorly differentiated, *CIN* conjunctival and corneal intraepithelial neoplasia, *CIS* conjunctival and corneal in situ carcinoma, *PNI* perineural invasion, *VAF* variant allele frequency, *TSG* tumor suppressor gene, *KNSTRN* kinetochore localized astrin binding protein coding gene, *Mb* megabase, *PFS* progression-free survival, *HNSCC* head and neck squamous cell carcinoma, *CNA* copy number alteration, *RTK* receptor tyrosine kinase, *PI3K* phosphoinositide 3-kinase.

CSCC and precursor lesions are mostly seen in elderly patients, aged, sun-exposed skin, which has been demonstrated to be an extended mosaic of multiple clones, predisposed to acquire further, potentially transforming events^8^. This has been reasoned both due to a temporally increased cumulative exposure to UVR, but also by the finding, that NER capacity significantly reduces with age, resulting in an increasingly inefficient DNA repair machinery^8,9^.

Besides UVR, many other factors contributing to the carcinogenesis of cSCC have been recognized. Discussion of all factors would be beyond the scope of this perspective. However, an important factor to mention here is immunosuppression: Immunosuppressed patients, especially solid organ transplant recipients (SOTR), are a considerable group with a 100-fold increased risk of developing aggressive cSCC^10,11^. Current data suggest a similar tumor mutational burden (TMB) of cSCC in immunocompromised versus immunocompetent patients^12^. However, there is evidence of distinct alterations, such as a higher frequency in HRAS mutations and a mutational signature related with exposure to azathioprine treatment^13,14^. This indicates that advanced cSCC arising upon immunosuppression may have an altered mutational landscape compared to advanced cSCC arising predominantly from UVR. The molecular biological identification of driver or druggable mutations and the development of a tissue- or liquid biopsy-based assay for its stratification would be a desirable advancement, especially in a setting of SOTR, in which targeted alternatives to immune system promoting immunotherapy could help circumvent difficult medical situations.

### Mutational landscape of cSCC

As UVR leads to a high mutational burden, cSCC is characterized by a diverse mutational landscape. Tissue- and liquid biopsy-based diagnostic approaches may depend on a thorough understanding of driving mutational events. In this perspective, we therefore conceived an up-to-date overview of selected reports on the somatic mutational landscape of cSCC and their contribution to the field (Table 1). A recent meta-analysis including 105 cSCC samples conceived the up-to-date most comprehensive list of 30 bona fide driver genes^15^. At this point, we do have considerable insight into the mutanome of cSCC and precursor lesions. We even have an idea of temporal significance of respective mutations. Unfortunately, we do not know in detail yet, which events become vital for transformation into invasive cSCC, aggressive disease or metastatic progression, after all.

As an example, TP53 and NOTCH1 are among the most frequently mutated genes in cSCC, but these mutations do not seem to drive clonal growth beyond a certain size without additional genetic, epigenetic or environmental contribution^6^. Therefore, one central question is, which mutation is the result of genetic drift or selection respectively. Largescale studies shedding light on temporospatial heterogeneity are therefore needed. This could be realized with upcoming technologies like single cell sequencing or spatial transcriptomics. With these methods only recently a tumor-specific keratinocyte (TSK) population localized to the leading edges of cSCC could be identified, which, in conjunction with basal and adjacent stromal and immune cells, exhibited invasive and immunosuppressive features^16^. Further transcriptomic, proteomic and metabolomic studies recently highlighted important correlations for cSCC and precursor lesions^17–19^. Particularly noteworthy in context on advanced cSCC, Shapanis et al. reported a significant correlation of poor outcome with increased annexin A5 (ANXA5) and dolichyl-diphosphooligosaccharide-protein glycosyltransferase noncatalytic subunit (DDOST) expression in a retrospective study of patients, which exhibited metastatic disease up to 5 years after primary surgery of cSCC^20^. They developed a prediction model of ANXA5 and DDOST showing higher sensitivity and specificity than cSCC clinical staging systems for estimating likelihood of metastases in cSCC^20^.

Another perspective is the identification of targetable events in the mutational landscape of cSCC. Jones et al. described a patient who experienced disease progression while on nivolumab treatment^21^. Based on a detected ERBB3 mutation, lapatinib was added to nivolumab resulting in stabilization of disease^21^. However, if frequent NOTCH1 loss-of-function mutation would be targeted, Martincorena et al. realistically stated that we might successfully treat 60% of cSCC, but with a considerable collateral damage to physiologically normal skin^7^.

Together, there is strong evidence that, for a deeper understanding of inter- and intrapersonal moieties in cSCC and subsequently the advancement of diagnostics and therapeutic decisions, identification of composite biomarkers rather than single biomarkers could picture the sophisticated interplay between tumor genetics, tumor mutational environment (TME), and other host factors.

## Current standard of diagnostics and treatment of advanced CSC

### Current diagnostic concept of advanced cSCC

**Fig. 1:**
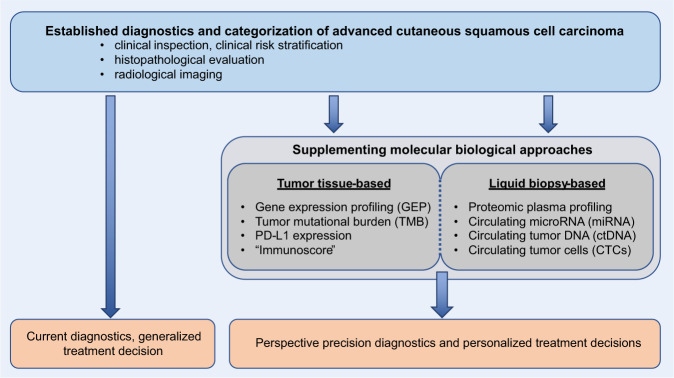
Current and perspective diagnostic concepts in advanced cSCC. Schematic illustration of current diagnostic key elements and perspective supplementary value of tumor tissue- and liquid biopsy-based molecular biological approaches.

Cutaneous manifestations of advanced cSCC are primarily investigated clinically (Fig. 1). Important clinical features, also for the later decision on further diagnostics and on therapeutic treatment regimen, are the localization of disease, palpable regional lymph nodes and the presence of often multiple co-existing lesions. Whole skin examination is therefore essential. Dermoscopy can help to further establish diagnosis. The tumor diameter is a distinguishing factor for classification into T-category in the American Joint Committee on Cancer’s (AJCC) staging system^22^. However, metastatic disease does not necessarily need to be clinically apparent as a large tumor.

Histopathological assessment of the lesion is a central component in advanced cSCC diagnosis (Fig. 1). Even if the tumor appears to be surgically unresectable, a tissue biopsy currently appears vital for confirmation of diagnosis of advanced cSCC. Besides the histopathological subtype of disease, high-risk features such as perineural invasion, low grade of differentiation, bone invasion, tumor thickness, and invasion beyond subcutaneous fat are important for stratification of patients^23^. At the presence of risk factors or clinical suspicion, lymph node sonography is performed for evaluation of locoregional metastases^24^. Further radiological assessment via computed tomography (CT) and cranial magnetic resonance imaging (cMRI) is sought in cases of suspected metastasis or suspicious findings in sonography (Fig. 1)^24^. Upon identification of metastatic foci, histopathological verification is desired, if surgically achievable.

Confirmed advanced cSCC cases are further discussed in multidisciplinary tumor boards for evaluation of a subsequent treatment regimen^25^. However, further tissue- or blood-based molecular characterization is not routinely performed due to a lack of reliable molecular prognostic and predictive biomarkers.

### Current treatment options for advanced cSCC

Although the vast majority of cSCC are successfully eradicated by surgical complete excision with excellent prognosis, aggressive cSCC subgroups are associated with a higher likelihood of recurrence, metastasis or even death^1^. In contrast to early cSCC, unresectable and metastatic disease is rare, but potentially life-threatening. Besides local radiotherapy, multiple strategies like chemotherapy (platin-based), targeted therapy (cetuximab) or immunotherapy (interferon) have been explored. Unfortunately, overall results were not encouraging, leaving advanced cSCC patients a rather dismal prognosis. However, PD-1 checkpoint inhibitor cemiplimab has been demonstrated a milestone in advanced cSCC treatment just recently: In open-label nonrandomized Phase-II pivotal EMPOWER-cSCC-1 trial, cemiplimab induced response rates of 47%, which led to FDA approval in September 2018 and EMA approval in June 2019^26^. In June 2020, pembrolizumab was approved for treatment of advanced cSCC by FDA, based on KEYNOTE-629 trial, underlining the rapidly increasing role of immuno-oncology in the therapeutic spectrum of cSCC^27^.

In general, current diagnostic standard of care in cSCC is already considerably efficient and easy to perform in comparison to tumor entities of internal organs, for example. However, in terms of advanced cSCC and especially prediction upon systemic treatment, this might not be the fact yet: although cemiplimab became a first-line gold standard treatment in advanced cSCC early after approval, up to 50% of the patient collective might not profit long-term. This is presumably a result of a precision medicine diagnostic gap of current standard of care diagnostics. In contrast to other entities like for example melanoma, there are only few reliable prognostic or predictive biomarkers. In the following, we will therefore discuss both the current status and possible future development and challenges of selected tumor tissue- and liquid biopsy-based diagnostic molecular biological techniques in advanced cSCC.

## Emerging diagnostic approaches in advanced CSCC

### Liquid biopsy-based approaches

There are potential advantages to blood-based liquid biopsy over tissue-based techniques to monitor tumor development during immunotherapeutic treatment. Blood can be easily drawn serially in a minimally invasive and reproducible manner, paving the way for optimal cancer surveillance^28,29^. Liquid biopsy may also provide a platform of real-time monitoring of tumor heterogeneity and residual tumor load and holds the potential for development of personalized therapeutic regimens^30,31^.

### Proteomic plasma profiling

Circulating proteins may be easily isolated from blood with the advantages of high sensitivity and easy standardization^28^. Up to date, few reports on proteomic plasma profiling (PPP) in cSCC have been presented^32,33^. However, besides an often limited stability, proteins are frequently not specific for cancer^28^. The combination of circulating tumor DNA (ctDNA) and PPP to form a composite biomarker may provide the advantage of increased specificity and sensitivity. Cohen et al. reported on CancerSEEK, a blood-based test, in which both ctDNA and PPP are combined and have been evaluated on eight surgically resectable cancer entities^34^. CancerSEEK was demonstrated to identify patients even with low tumor burden or at early stage of disease with sensitivities of ~70–98%^34^. Unfortunately, cSCC was not included in this test. Part of this reason may be the frequency of cSCC in the population and proportionally fewer rapidly advancing cases as compared to breast cancer or lung cancer. Further, circulating proteins used in the CancerSEEK panel were previously found to be specific for certain entities. No specific circulating protein has been identified for advanced cSCC so far, possibly making combination for ctDNA and PPP difficult in this case. This may in turn be the consequence of cSCC not yet being in focus of the latter studies, as described above. Thus, further reports on PPP and ctDNA analysis in advanced cSCC are required. In the authors’ opinion, PPP in combination with ctDNA or circulating tumor cell (CTC) analysis might exhibit potential for future development of a composite biomarker panel in advanced cSCC.

### Circulating microRNA

MicroRNAs (miRNAs) are endogenous, small non-coding RNAs, which may control basal cell biological processes via post-transcriptional regulation of gene expression^35^. Dysregulation of miRNA expression is involved in most cancer hallmarks such as regulation of cell apoptosis, invasion, proliferation, or migration^36^. For cSCC, a plethora of differentially regulated miRNAs with important roles in formation and progression of disease have been identified. These have been thoroughly reviewed recently^37,38^. MiRNAs may be detected in peripheral blood and other body fluids^39,40^. Extracellular vesicles may contain considerable miRNA amounts^41^. Their stability in blood makes them potential candidates for valuable biomarkers^42,43^. In cervical SCC different miRNA patterns have been identified predictive biomarkers for lymph node metastasis in early-stage disease^44^. In another cohort of 79 cSCC patients, prognostic sub-groups based on miRNA-203 and miRNA-205 expression patterns could be defined^45^. However, a more profound knowledge on miRNA signatures as biomarkers for monitoring therapeutic responses upon immunotherapy of advanced cSCC will be important for translation into clinic.

Besides investigations on the utility of miRNAs as a supplemental biomarker in advanced cSCC, both topical and systemic miRNA targeting strategies such as introduction of defective miRNA or blocking of miRNA overexpression (antagomiRs, RNA inhibitors) have been presented and are subject to futher investigations in cSCC^46,47^.

### Circulating tumor DNA

The mutant fraction of cell-free DNA representing tumor cell-derived DNA circulating in blood is termed ctDNA. It is released by apoptotic and/or necrotic tumor cells and mostly studied in plasma, but can also be isolated from other body fluids, such as saliva^48,49^. It is easy and well-established isolation as well as detection of also rare mutations delineate ctDNA a promising candidate not only for prognostication of advanced CSCC, but also monitoring of disease as well as the detection of minimal residual disease^28^. Depending on the status of disease and tumor burden, sensitivity may be limited. However, multiple assays like digital droplet PCR, modified next generation sequencing (NGS), standard NGS or sanger sequencing are available with high differences in sensitivity, but also considerable differences in cost and turn-around time for each sample to be analyzed^30^.

As for cSCC, ctDNA analysis holds a potential which has not been exploited so far. In contrast, for head and neck squamous cell carcinoma (HNSCC), ctDNA could be detected in several studies demonstrating feasibility already^50–53^. Latest results in this tumor entity revealed a correlation of ctDNA quantity and tumor burden, the prediction of lymph node metastasis and overall survival by a copy number instability score (CNI), making ctDNA a promising candidate biomarker for molecular diagnostics^50,53,54^. A so far unique demonstration of detection of HPV ctDNA in oral HNSCC clearly associated pre- and post-treatment ctDNA levels from blood and saliva with treatment success or failure and identified disease recurrence early after treatment^55–58^. Moreover, Cabel et al. provided a first insight in this regard into anal SCC by demonstrating detectability of HPV ctDNA and post-treatment association with poor outcome^55^. Thus, future ctDNA based investigations on cSCC, especially in previously HPV-associated locations, should be intiated.

As mentioned above and in contrast to HNSCC, for most cSCCs a high TMB due to cumulative UV-exposure is characteristic. Additionally, only few actionable driver mutations are known, resulting in the current challenge of ctDNA detection against the background of intratumoral mutational heterogeneity. Compared to HNSCC, molecular data on cSCC is rather rare in publicly available databases. Therefore, defining ctDNA libraries and ctDNA panels for routine diagnostics will be crucial for success of ctDNA analysis in cSCC. Besides these current pressing action points in cSCC there are several unanswered key questions such as interpretation of ctDNA detectability and origin in the considerable cohort of patients with multiple cSCC or field cancerization respectively (Box 1). Differentiability of ctDNA between different cSCC sites and in metastasized cases re-traceability back to the originating cutaneous site are other important questions (Box 1).

Box 1 Projected challenges in use of liquid biopsy in advanced cSCC - A high TMB and multiple candidate driver mutations characterize cSCC. Which alterations can be diagnostically addressed? Preparation of libraries and panels of ctDNA will presumably prove more suitable than single probes.

- Against the background of multiple frequent mutations, detection of seldom mutations might be of relevance to monitor clonal heterogeneity, e.g. upon treatment. Sensitivity of ctDNA and CTC is a common issue and has to be evaluated in the latter regard.

- Field cancerization or the co-occurence of multiple cSCC sites might confound the picture of the lesion of interest. Could tumor-derived material such as ctDNA be released into the blood stream both by the primary tumor of interest leading to advanced disease and secondary tumors? Other epithelial cancers have been successfully identified by liquid biopsy at an early stage previously. A realistic underlying mechanism could therefore hypothetically be that detectable ctDNA was measurable already in cases of localized invasive cSCC. In that case, a subsequent challenge could be traceability of liquid biopsy signals back to the ”causative“ primary tumor leading to advanced disease. One possible attempt could be mutational profiling of the respective tumor tissues combined with ctDNA panel analysis. It could be speculated that this approach might be too costly and laborious for later implementation into routine clinical diagnostics of advanced cSCC cases.

- Detectability of CTCs by label dependent or independent techniques in advanced cSCC? Using label dependent techniques, for example, feasibility of CTC detection will rely on cellular markers on CTC. As cSCC is from epithelial origin, established techniques using epithelial markers could be a potential approach.

### Circulating tumor cells

CTCs are intact viable tumor cells released by primary tumors or metastatic tissue into the blood at low concentrations. They have been used in clinical studies for real-time monitoring of tumor evolution under immunotherapy in tumor entities like melanoma^59^. For cutaneous HNSCC, a pilot study including ten patients with regional metastatic disease successfully identified CTCs in 80% (up to 44 cells/9 ml blood)^60^.

Besides CTC enumeration, characterization of immune-marker expression on CTCs is an emerging field^61^. Although a meta-analysis of pre-treatment PD-L1 CTC expression in HNSCC did not reveal significant associations with progression-free survival, Strati et al. reported that increased PD-L1 expression on CTCs in locally advanced HNSCC was an independent prognostic marker of decreased overall survival and progression-free survival after radio-chemotherapy^62–65^. Currently, a couple of studies are further investigating immune-marker expression on CTCs and their correlation with tumor tissue markers or blood-based markers in HNSCC^66,67^. However, cSCC remains to be investigated in regard of CTC characterization (Box 1).

### Tumor tissue-based approaches

Tumor tissue-based analyses are limited to primary and, if applicable, metastatic tumor tissue. It may prove difficult to identify the primary tumor site responsible for metastatic disease in patient cases most frequently associated with multiple precursor lesions or even parallel cSCC lesions. Additionally, repetitive analyses remain challenging to perform as invasive procedure is involved and to be justified taking operative risks into consideration. Therefore, tumor tissue-based principles frequently do not hold the option for monitoring under systemic treatment when primary tumors have been excised and metastases are hard to biopsy. Another important challenge is that a high tumor heterogeneity may not be representatively projected in a mere biopsy. However, it should be considered, that tissue-based diagnostics is part of standard of care in cSCC already, and therefore tissue-based advancements in the field could more rapidly and easilier be implemented into routine diagnostics compared to liquid biopsy-based approaches. In the following, a selection of tumor tissue-based principles is discussed.

### Tumor mutational burden

TMB is a biomarker measuring total counts of somatic mutations per megabase of the investigated tumor genome^68^. High TMB has recently been correlated with favorable outcome of immune checkpoint inhibitor (ICI) treatment in tumor entities like melanoma^69^. The current perspective is that a high TMB may result in a high neoantigen load, which may lead to increased T-cell activity, thus enhancing anti-tumor response^70^. Based on the assumption of a correlation between TMB and overall response rate, cSCC has therefore been previously projected to exhibit among the highest response rates to anti PD-1 treatment^71^. However, advanced cSCC is comparatively rare and therefore might be often not included in large studies on TMB or represented by a fairly small sample size. Additionally, recent findings indicate that the effect of a high TMB depends on the treatment context, respectively on prior ICI treatment and type of ICI regimen^69,72^. Larger studies on the prognostic and predictive value of TMB regarding ICI treatment in advanced cSCC should be striven for. Moreover, several limiting barriers for TMB to be adopted as a biomarker into clinical practice yet have to be overcome^73^. Of these, a lack of harmonization of applied methods to investigate TMB across studies, adequate methods to convert TMB estimates across different panels and missing of robust predictive cut-offs are considered the central limitations^73^.

### PD-L1

The expression of PD-L1 on tumor cells has been associated with poor prognosis due to promotion of an immunosuppressive TME in many cancer entities. However, an inverse correlation has been reported between PD-L1 expression and poor prognosis in long-term follow-up analyses. For cSCC, PD-L1 expression has been associated with an increased risk of metastatic disease and the presence of high-risk pathological findings^74,75^. However, other studies did not find correlation of PD-L1 expression with prognostic features in cSCC^76^. This illustrates the current limitations for clinical use of PD-L1 as an exclusive biomarker in advanced cSCC, although it might be determined a valuable marker in a composite panel. Further studies with larger cohorts are needed to understand and confirm whether testing for PD-L1 aids in cSCC prognostication or prediction upon ICI treatment. Heterogeneity in findings may be reasoned by a considerable methodological and definitional variability among conducted studies^77^.

### Immunoscore

The extent of tumoral spread serves as a standard parameter for classification of patients into stages. This categorization of patients revealed heterogeneous prognostic populations within staged subgroups^78^. Cancer is increasingly defined as a complex interplay between the tumor and the host’s immune system^78^. In 2006, Galon et al.^79^ demonstrated in situ-analysis of infiltrating adaptive immune cells to be a valuable prognostic tool in colorectal cancer patients. They introduced “Immunoscore” as a method assessing type, density and location of in situ-T-cell infiltrates within colorectal tumor tissue and discovered a prognostic superiority to mere classification of patients regarding the extent of tumoral spread^79^. The concept of this immune texture is based on the dependency of tumor immune microenvironment interplaying with the efficacy of an immunotherapeutic treatment. Based on an individual’s immune cell signature, treatment outcome is sought to be predicted. Immunoscore data on cSCC has not been published so far. The authors see an applicability of this technique for future cSCC prediction to immunotherapeutic response critically: cSCC inherently exhibits a high mutational load. As described above, this is considered a favorable feature in terms of immunotherapeutic addressability. However, it may also reflect tumor heterogeneity taking reports on multiple clones even within one primary tumor into consideration^7^. Furthermore, this tumor-based technique would demand combination with another approach allowing for identification of the primary cSCC site relevant for advanced disease at a clinical coincidence of other cSCC tumor sites.

### Gene expression profiling

In the field of histology and histopathology, chemical analysis of tumor tissue, mostly from FFPE samples, is considered diagnostical standard to accurately define a cancer entity and tumor subtypes. In order to more precisely define tumor subgroups on an individual basis, gene expression profiling is an increasingly used technique to molecularly characterize tumor tissue. For example, gene expression of tumor tissue may be quantified by DNA microarray or high-throughput real time-PCR (RT-PCR) with RNA from FFPE samples. Differentially expressed genes may be correlated with clinical impact on parameters like therapeutic responsiveness or response prediction. Such analyses allow for composition of gene expression profiles (GEP) or definition of signatures that may be further evaluated as clinical biomarkers in prospective trials.

Ioannidis et al.^80^ identified potential candidate genes and susceptibility loci of cSCC compared to non-cSCC controls via GEP analysis. This and other recent reports demonstrate the potential of GEP for defining cSCC biomarkers and understanding molecular pathogenesis of cSCC^17^.

Recently, a promising 40-gene expression profile test (DecisionDx-SCC; Castle Biosciences, Inc.) to predict metastatic risk in localized high-risk cSCC has been presented that could complement current staging systems for patients with high-risk cSCC^81^. It should be noted that there is not a clear recommendation for the optimal timing of testing yet^82^. As GEP testing is now available in cSCC, an expert panel has recently underlined the concern of early incorporation into clinical routine until definitive results of prospective trials become available^82^. It is therefore currently recommended for use as an additional data point instead of a surrogate for standard of care diagnostics or treatment^82^.

## Conclusion

The majority of cSCC cases are considered localized disease and current standard-of-care screening for localized cSCC and antecedent lesions is well-established, easy to perform and cost-efficient. Of note, a considerable cohort of inoperable and metastatic cases can be deducted from a hardly subsumable yet rising incidence of cSCC cases worldwide. In contrast to localized disease, current diagnostics of advanced cSCC does not reflect the clinical precisional medicine need in the era of immunotherapeutic treatment. Recent studies addressing molecular biology of advanced cSCC have set the basis for a broader knowledge on the mutational landscape of this tumor entity. Despite recent scientific advances, it remains difficult to identify significant and actionable driver mutations of advanced disease. Reliable biomarkers for prognostication, monitoring and characterization of disease are a desired, not yet clinically realized need in advanced cSCC. It will be important to implement state-of-the art techniques into routine diagnostics of advanced cSCC to address current challenges like detection of disease or monitoring treatment response in order to further personalized medicine. To achieve this goal, diagnostics should not only focus on tissue-based analysis. Liquid biomarker analysis of CTC, ctDNA, miRNA, or PPP in combination with other techniques hold a, partly unexploited, potential in advanced cSCC and should become a central part of future investigations. The development of novel biomarkers is most likely to supplement the established diagnostic standard rather than substituting it (Fig. 1).

## Data Availability

Data sharing not applicable to this article as no datasets were generated or analyzed during the current study.
